# An overview of Volume 14, No 1 (2022)

**DOI:** 10.4102/jamba.v14i1.1379

**Published:** 2022-12-21

**Authors:** Jason K. von Meding

**Affiliations:** Florida Institute for Built Environment Resilience (FIBER), University of Florida, Gainesville, United States of America

Welcome to Volume 14, No. 1 of *Jàmbá: Journal of Disaster Risk Studies* (*Jàmbá*)! As a new Associate Editor, it has been a privilege this year to become part of the process of managing this wonderful open-access disaster journal. In this piece, I’d like to briefly frame the contents of this issue and offer some thoughts on reading across the original research articles that we have published. The articles in this issue cover a broad geography, with findings from Pakistan, Zimbabwe, Indonesia, Uganda, Iran, Ethiopia, Nigeria, Madagascar, the Philippines and South Africa. This speaks to *Jàmbá*’s commitment to feature Global South authors and of course readers, delivering cutting-edge open access science on disasters to the communities that are most impacted by them. The collection features 41 Original Research articles, 2 Opinion Papers and a Book Review.

We know that risk is understood and experienced differently depending on context and identity, and this collection captures the diversity of perspectives that need to be considered; from people with disabilities (Ssennoga et al. 2022), to pastoral households (Abrham & Mekuyie 2022), and farmers (Dibakoane, Siyongwana & Shabalala 2022; Matimolane et al. 2022), to students (Kutywayo et al. 2022), to families that include older people (Fatmah 2022). Various professionals also contribute to managing risk in society, and this is explored in relation to educators (Dzvimbo et al. 2022), social workers (Matlakala, Makhubele & Nyahunda 2022), librarians (Chisita & Ngulube 2022), first responders (O’Neil & Kruger 2022), and health care professionals (Capili et al. 2022). The differential experience of risk is invariably shaped by historical legacies of injustice and inequality, as Magana and Suso (2022) point out with regards to the impact of colonial land policies in Zimbabwe.

In the face of this unequally distributed risk, there is strong evidence presented in this collection as to the efficacy of community-centred approaches to both risk management and research about risk management. Nkombi and Wentink (2022) investigate public participation in Disaster Risk Reduction (DRR), while Chinguwo and Deus (2022) show how more resources must be allocated for community-based early warning systems, and the disconnect with more regional and national systems resolved. Communities often receive outside support, and Shah et al. (2022) explore inter-agency collaboration.

It is clear that the benefits of community-centered research and practice are many, and the outcomes can be radical and transformative – as well as more grounded in local realities. Purwitaningsih et al. (2022) explores the benefits and limitations of participatory flood inundation mapping, while Paripurno et al. (2022) share beneficiary perspectives on the importance on community-driven livelihood recovery. Aksa and Afrian (2022) discuss the importance of ‘gotong royong’ (mutual cooperation) as social capital in coastal adaptation. These articles demonstrate the necessity of close relationships, trust and solidarity between all stakeholders concerned with disaster risk management, across scales and geographies. This would help to resolve persistent problems – such as food insecurity – felt at the local level by underserved and isolated communities (Matunhu, Mago & Matunhu 2022; Ngwenya, Lunga & Van Eeden 2022).

Given that disaster risk management is a priority in policy and governance, we are glad to see several articles analysing the degree to which our systems of care are succeeding in reducing risk and planning for the future. The collection features analysis of multi-issue policy coherence (Zembe, Nemakonde & Chipangura 2022), institutional ecosystems (Ichsan 2022; Lassa, Nappoe & Sulistyo 2022), resilience planning processes (Ghafuri & Koohpaei 2022; Nkamisa et al. 2022; Matshusa & Leonard 2022; Raharjo, Sarjana & Safitri 2022; Terblanche, De Sousa & Van Niekerk 2022; Atanga & Tankpa 2022) economic sustainability (Mavhura & Aryal 2022; Isa & Mardalis 2022), education (Aiyub Kadir & Nurdin 2022, Dimitrova & Mokhele 2022) and communication (Dehghani et al. 2022), overall development agendas (Mthembu & Nhamo 2022; Elum & Lawal 2022), insurance (Mushonga & Mishi 2022) and the integration of Sustainable Development Goals into risk management (Louw & Esterhuyzen 2022).

As we continue to struggle through the lasting impacts of the COVID-19 pandemic, John et al. (2022) contribute a scoping study of how rural vulnerabilities have been impacted, while Khowa, Cimi and Mukasi (2022) look more closely at the impact of lockdown regulations. An institutional approach to working with communities through pandemic response is contributed by Leeney et al. (2022), showing that the key to effective operational response was local partnership and presence in communities.

I hope that you will enjoy reading this collection of original research articles and consider submitting your own work to a future issue. We also value alternative submission types, and invite you to enjoy Alice Ncube’s (2022) extensive review of *The Continuing Storm: Learning from Katrina* by Kai Erikson and Lori Peek as well as the Opinion Papers by Aksa (2022) and Matandirotya (2022). *Please share this with your networks*. Everyone at *Jàmbá* appreciates your support of our efforts to bring you outstanding open access research from the Global South.
